# Grim-19 deficiency promotes decidual macrophage autophagy in recurrent spontaneous abortion

**DOI:** 10.3389/fendo.2022.1023194

**Published:** 2022-10-25

**Authors:** Yang Yang, Haoran Liu, Yue Zhao, Chen Geng, Lan Chao, Aijun Hao

**Affiliations:** ^1^ Key Laboratory for Experimental Teratology of Ministry of Education, Department of Anatomy and Histoembryology, School of Basic Medical Sciences, Cheeloo College of Medicine, Shandong University, Jinan, China; ^2^ Center for Reproductive Medicine, Department of Obstetrics and Gynecology, Qilu Hospital of Shandong University, Jinan, China

**Keywords:** GRIM-19, recurrent spontaneous abortion, macrophage, autophagy, proinflammatory cytokines

## Abstract

Dysregulation of decidual macrophages leads to the occurrence of recurrent spontaneous abortion (RSA). However, the role of macrophages in RSA occurrence remains unclear. In this study, we found that the expression of Grim-19 was decreased, and the expression of autophagy related proteins Beclin1, LC3B II/I and BNIP3 was markedly upregulated in decidual macrophages of RSA patients compared with the normal pregnancy group. Furthermore, we demonstrated that downregulation of GRIM-19 increased the expression of autophagy related proteins Beclin1, LC3B II/I, BNIP3 and the proinflammatory cytokines IL1B, IL6 and TNFa in uterine mononuclear cells of *GRIM-19^+/-^* mice. The proportion of CD45+CD11b+F4/80+LC3B+ cells in *GRIM-19^+/-^* mouse uteri was significantly higher than that in WT mouse uteri. In addition, we confirmed that inhibition of *Grim-19* by siRNA enhanced the expression of autophagy related proteins in RAW264.7 cells and THP-1 cells. More importantly, downregulation of *Grim-19* in RAW264.7 cells promoted the release of proinflammatory cytokines and promoted phagocytic activity, which could be reversed by autophagy blockade. For THP-1-derived macrophages, the results of RNA-seq suggested that Grim-19 mainly modulates immune and inflammatory-related pathways, leading to cytokine production, and thus contributing to inflammation. Therefore, our data reveal that Grim-19 deficiency influences macrophage function, characterized by enhanced proinflammatory cytokines and phagocytic activity, and this might be regulated by autophagy. This may represent a novel mechanism for the occurrence of RSA.

## Introduction

Recurrent spontaneous abortion (RSA) affects approximately 5% of childbearing age women health, and it is defined as two or more consecutive spontaneous miscarriages occurring before 20 weeks of gestation (1). Established risk factors are abnormal endocrine functions, genetic abnormalities, uterine malformations, selected maternal autoantibodies, and immune imbalance (2). However, the cause of RSA remains largely unknown and further research is needed.

Current research shows that successful pregnancy depends on the maintenance of maternal-fetal immune tolerance. Decidual immune cells constitute the basis of maternal-fetal immune tolerance, and mainly composed of macrophages, natural killer (NK) cells and T cells (3). Macrophages constitute 20-30% of total decidual leukocytes in early pregnancy and are important cells in the innate immune system. Functions of macrophages include regulating inflammatory responses, clearing cellular debris, and protecting cells from pathogens (4, 5). Macrophages play important roles in homeostatic and tolerogenic properties, and decidual macrophage dysregulation contributes to the development of RSA (6). However, the role of macrophages in the pathogenesis of RSA remains unclear.

Macrophages maintain metabolic homeostasis through cytokine signaling regulation and macrophagic phagocytosis. Recent studies have begun to elucidate the intrinsic linkage between autophagy and macrophage function (5). Although, autophagy deficiency in macrophages has been involved in several inflammatory diseases, such as arthritis and systemic lupus erythematosus (SLE), the role of autophagy in macrophage inflammatory cytokine production remains unclear. Defects in macrophage autophagy may contribute to inflammatory diseases, such as reduced macrophage autophagy leading to liver inflammation and progression of liver injury (7). However, excessive autophagy leads to autophagic death of macrophages and further aggravates the inflammatory response (8).

Grim-19 (gene associated with retinoid-interferon-induced mortality 19) has been identified as a subunit of mitochondrial complex I and plays important roles in electron transfer activity (9). Downregulation of Grim-19 has been observed in multiple tumors, and aberrant expression of Grim-19 is involved in apoptosis and cell growth inhibition (10). Recent studies have identified important functions of GRIM-19 in regulating immune responses and inflammation. GRIM-19 was shown to augment regulatory T (Treg) cell differentiation, while suppressing T helper 17 (Th17) cell differentiation, by inhibiting signal transducer and activator of transcription 3 (STAT3) phosphorylation (11). Additionally, a recent study also reported that Grim-19 contributes to the reduction of invasion in a hypoxic environment by repressing autophagy in human colorectal cancer (12).

In this study, we detected the expression of Grim-19 in decidual macrophages isolated from women with normal pregnancies and patients with RSA. Our data suggested that Grim-19 is downregulated in decidual macrophages of RSA, and aggravates RSA by promoting macrophage autophagy and the inflammatory response.

## Materials and methods

### Patients and tissue samples

This study has been reviewed and approved by the Medical Ethics Committee of Qilu Hospital of Shandong University (Number of approval: KYLL-2017(KS)-144). Informed consent was obtained from all human subjects. All the study subjects were recruited from the department of gynecology and obstetrics, Qilu Hospital of Shandong University.

A total of 10 women with normal pregnancy (NP) who opted to terminate pregnancy for nonmedical reasons and 10 women with RSA who had experienced at least two consecutive spontaneous early miscarriages (6-12 weeks) were enrolled. The patients with RSA showed no statistical difference in gestational ages (8.00 ± 0.58 weeks) and ages (32.80 ± 1.23 years) when compared with normal pregnant women (7.60 ± 0.64 weeks, 29.60 ± 1.14 years). None of the RSA subjects had any risk factors such as endocrine or metabolic disorders, autoimmune diseases, anatomic abnormalities, infections, and paternal or maternal chromosomal abnormalities.

Decidua tissues were collected during operation and immediately sent to the laboratory. Decidua tissues were grinded into cell suspension. Human mononuclear cells were separated from decidua cell suspension by gradient centrifugation on Ficoll-Hypaque (Lymphoprep, AXIS-SHIELD PoCAs, Oslo, Norway) at 800g for 30min at room temperature. CD14+ macrophages were selected from mononuclear by CD14+ magnetic beads (Miltenyi Biotec, Germany).

### Animals

*GRIM-19^+/-^* mice (C57BL/6J) were obtained from model animal research center of Nanjing university. *Grim-19* knockout model was created by CRISPR/Cas9-mediated genome engineering. Transcript ENSMUST00000110167.4 has 5 exons, with the ATG start codon at exon 2 and TAG stop codon at exon 5. Specific gRNAs in intron 2-3 and intron 3-4 were designed, which direct Cas9 endonuclease cleavage *Grim-19* gene and create a double-strand break. Such breaks were repaired by non-homologous end joining, and *Grim-19* gene was disrupted by deletion of exon3 (cagagactgccctggggaagtggctgaggaagga—227bp—GGTGGTGAGC aggggttctgtgaggatgagggaactcctac). Agarose gel electrophoresis was carried to verify the genotype of the mice, which using DNA extracted from the tip of tail. The following primer sequences were used: Forward: 5’CCACCCCCAAGTGTAAAACT

ATC3’, and Reverse: 5’GCACAGGCAGGCAATAGCAG3’.

Mice were maintained in the barrier facility at the Shandong University with free access to sterile reverse osmosis water and irradiated laboratory food. The cages and bedding were changed weekly to keep the environment stable. For all the experiments, 8- to 10-week-old female mice were used. All procedures of the animal experiments were approved by the Ethical Committee of Shandong University. All efforts were made to minimize suffering of mice.

### Isolation of mouse uterine mononuclear cells

*GRIM-19^+/-^* and wildtype (WT, littermates to *GRIM-19^+/-^* mice) mice were inspected every morning for vaginal plugs. The day when a plug appeared was designated as day 0 of pregnancy. Mice were sacrificed on day 11 of pregnancy, and mouse uterine tissues were collected. Unpregnant mice were excluded. Mouse uterine tissues were dissected into 1-3 mm pieces. Single-cell suspensions were prepared by Lamina Propria Dissociation Kit (Miltenyi Biotec, Germany) following the manufacturer’s instructions. Mononuclear cells were isolated by using Percoll Gradients (Solarbio, Beijing, China) density gradient centrifugation at 300g for 20 min.

### Flow cytometry

A total of 4 mouse uterine tissues (day 11 of pregnancy) were taken from both groups, and mononuclear cells were separated. Mononuclear cells were incubated with anti-mouse CD16/32 (Fc block) for 10mins at room temperature to prevent non-specific binding. Cells were then incubated for 30 mins at room temperature in the following antibody cocktails: CD45-percp-Cy5.5 (Invitrogen, California, USA, 45-0451-82), CD11b-FITC (Invitrogen, California, USA, 11-0112-86) and F4/80-APC (Invitrogen, California, USA, 17-4801-82). The cells were fixed and permeabilized with Perm/Fix solution (BioLegend, USA) and were then stained with LC3B-PE (Cell Signaling Technology, USA, #8899S) after the surface staining. Stained cells were washed twice with PBS and analyzed with a FACS Calibur flow cytometer. We first gated on cells (gate P1) using forward scatter (FSC) and side scatter (SSC), then gated on CD45+ cells (gate P2), then gated on CD11b+F4/80+ cells (gate P3), and analyzed LC3B+ cells in a CD11b+F4/80+ gate. All staining was performed in accordance with manufacturer’s protocols. Isotype controls were used to confirm antibody specificity. Single colour stain controls were used to enable correct compensation.

### Cell lines and culture

Murine macrophage RAW264.7 cells were obtained from the American Type Culture Collection. The cells were cultured in Dulbecco’s modified Eagle medium (DMEM, Gibco, Carlsbad, CA, USA) supplemented with 10% (v/v) fetal bovine serum (FBS) (Sigma-Aldrich, Saint Louis, MO, USA) and 1% (v/v) penicillin-streptomycin (Sigma-Aldrich, Saint Louis, MO, USA) in a water-jacketed incubator (Thermo, Wilmington, DE, USA) with 5% CO_2_ at 37°C.

Immortalized cells derived from the peripheral blood of a patient with acute monocytic leukaemia (THP-1) (TIB-202, ATCC) were cultured in Roswell Park Memorial Institute 1640 (Gibco, Carlsbad, CA, USA) supplemented with 10% (v/v) fetal bovine serum (FBS) (Sigma-Aldrich, Saint Louis, MO, USA) and 1% (v/v) penicillin-streptomycin (Sigma-Aldrich, Saint Louis, MO, USA) in a water-jacketed incubator (Thermo, Wilmington, DE, USA) with 5% CO_2_ at 37°C. THP-1 cells were treated with phorbol ester (PMA, Sigma, USA) (50ng/ml) for 48h to obtain THP-1-derived macrophages.

Knockdown *GRIM-19* by small interfering RNA (siRNA) (GenePharma Co., Ltd, China) was performed by Lipofectamine RNAiMAX (Thermo, Wilmington, DE, USA) according to the manufacturer’s instructions. The following sequences were used: GRIM-19, 5’-GGAUUGGAACCCUGAUCUATT-3’, and scramble, 5’-UUCUCCGAACGUGUCACGUTT-3’. The culture medium was replaced after 6 hours of incubation. In 3-methyladenine (3MA) group, cells were cultured with 5mM 3MA (Sigma-Aldrich, Saint Louis, MO, USA) for 12h. Then, 48 hours after transfection, the cells were collected.

### Western blot analysis

Protein was extracted in lysis buffer (Beyotime, China). Bicinchoninic acid (BCA) Protein Assay kit (Beyotime, China) was used to evaluate the protein concentration. Proteins were separated by sodium dodecyl sulfate-polyacrylamide gel electrophoresis (10%), and transferred to polyvinylidene difluoride (PVDF) membranes (BioRad, USA). The membrane was blocked with 5% skimmed milk at room temperature for 2h, and then incubated with primary antibodies against following proteins: anti-GRIM-19 (Abcam, UK, ab110240, 1:1000), anti-Beclin1 (Cell Signaling Technology, USA, #3495, 1:1000), anti-LC3B (Abcam, UK, ab192890, 1:1000), anti-BNIP3 (Abcam, UK, ab109362, 1:1000), anti-GAPDH (Abcam, UK, ab9485, 1:2000) overnight at 4°C. After washing with Tris-buffered saline Tween-20 (TBST), the membranes were incubated with horseradish peroxidase (HRP)-conjugated secondary antibody at room temperature for 1h (Zhongshan Golden Bridge Biotechnology Co., LTD., China). Enhanced chemiluminescence (ECL) reagents (Merck Millipore, USA) was used to detect immunoreactivity. Immunoreactivity was quantified by densitometry using ImageJ software (National Institutes of Health, USA).

### Quantitative real-time PCR

Total RNA was extracted from using Trizol reagent (Invitrogen, California, USA), in accordance with the manufacturer’s instructions. cDNA was synthesized by using a ReverTra Ace qPCR RT kit (TOYOBO, Osaka, Japan). The cDNA was amplified by the SYBR Green qPCR kit (TOYOBO, Osaka, Japan). β-actin gene expression was used for normalization. The following primer sequences were used: GRIM-19, Forward: 5’GGGGCCTTGATCTTTGGCTA3’, and Reverse: 5’AAGTCCTCAATCAGCAGGCG3’; IL1B, Forward: 5’GTGTCTTTCCCGTGGACCTT3’, and Reverse: 5’AATGGGAACGTCACACACCA3’; IL6, Forward: 5’CTTCTTGGGACTGATGCTGGT3’, and Reverse: 5’CTCTGTGAAGTCTCCTCTCCG3’; TNFa, Forward: 5’CGGGCAGGTCTACTTTGGAG3’, and Reverse: 5’ACCCTGAGCCATAATCCCCT3’; Beclin1, Forward: 5’AACCGCAAGATAGTGGCAGA3’, and Reverse: 5’CTCTCTGATACTGAGCTTCCTCC3’; LC3, Forward: 5’ATCGCGGACATCTACGAGC3’, and Reverse: 5’AGGTTTCCTGGGAGGCGTA3’; BNIP3, Forward: 5’TCAGCAATAATGGGAACGGG3’, and Reverse: 5’AGCTACTCCGTCCAGACTCAT3’; β-actin, Forward: 5’GGCTGTATTCCCCTCCATCG3’, and Reverse: 5’CCAGTTGGTAACAATGCCATGT3’. Relative gene expression levels were analyzed by 2^−ΔΔCT^ method.

### Transmission electron microscopy

The RAW264.7 cells after 48h transfection were harvested and fixed with 2.5% glutaraldehyde for 1h at 4°C. After fixation, cells were treated with 1% cacodylate-buffer osmium tetroxide for 4h at 4°C. Cells were dehydrated with graded alcohol and embedded. Ultrathin sections were cut and then stained by uranyl acetate and lead nitrate. Finally, the samples were observed with a JEM-1200EX transmission electron microscope (Japan Electronics and Optics Laboratory, Tokyo, Japan). Three randomly selected areas were obtained.

### Enzyme-linked immunosorbent assay

SiRNA was used to knockdown *Grim-19* in the RAW264.7 cells. In 3MA group, cells were cultured with 5mM 3MA (Sigma-Aldrich, Saint Louis, MO, USA) for 12h. Then, 48 hours after transfection, the culture supernatant were collected. ELISA was performed to detect the inflammatory factors IL1B, IL6 and TNFa in culture supernatant collected using commercial kits (SEA563Mu for IL1B; SEA079Mu for IL6; SEA133Mu for TNFa; Cloud-Clone Corp., Wuhan, China) following the manufacturer’s instructions.

### Phagocytosis assay

Twenty-four hours after transfection and treatment with 3MA, RAW264.7 cells were incubated with fluorescein isothiocyanate- (FITC-) dextran (1mg/mL, Sigma-Aldrich, Saint Louis, MO, USA) at 37°C for 1h. After incubation, the cells were washed twice with PBS, and the percentage of intracellular FITC-dextran was determined by Fluorescence Activating Cell Sorter (FACS).

### RNA-seq analysis

Total mRNA was extracted from control siRNA or *GRIM-19* siRNA treated THP-1 cells according to the manufacturer’s instructions (Thermo Fisher Scientific, USA). Total RNA samples were assessed for purity, concentration, and integrity prior to further analysis. The purified mRNA was fragmented and reverse transcribed to create the final cDNA library in accordance with the protocol for the mRNA-Seq sample preparation kit (Illumina, San Diego, USA). The cDNA libraries were then sequenced on an Illumina Novaseq™ 6000 (LC Bio Technology CO., Ltd. Hangzhou, China) according to the manufacturer’s instructions. The expression level of a certain gene was quantified as fragments per kilobase of transcript per million mapped reads (FPKMs). The differential gene expression was identified using DESeq2 software. The genes with the parameter of false discovery rate (FDR) below 0.05 and absolute fold change ≥ 2 were considered differentially expressed genes (|log_2_FC|≥1 & q<0.05). The volcano plot, heatmap, GO and KEGG enrichment analysis of differentially expressed genes (DEGs) were generated with OmicStudio tools with clusters Profiler R package (https://www.omicstudio.cn/index).

### Statistical analysis

The GraphPad Prism Version 7.0 (GraphPad Software, USA) statistical software program was used for statistical analysis. All experiments were independently repeated at least three times. Data are shown as the mean ± standard deviation (SD). Statistical analyses of more than two groups were performed using one-way analysis of variance (ANOVA) followed by Tukey’s *post hoc* test or an independent samples t test. *p*<0.05 was considered statistically significant.

## Results

### The expression of Grim-19 is decreased in decidual macrophages of RSA patients and is negatively correlated with autophagy

**Figure 1:**
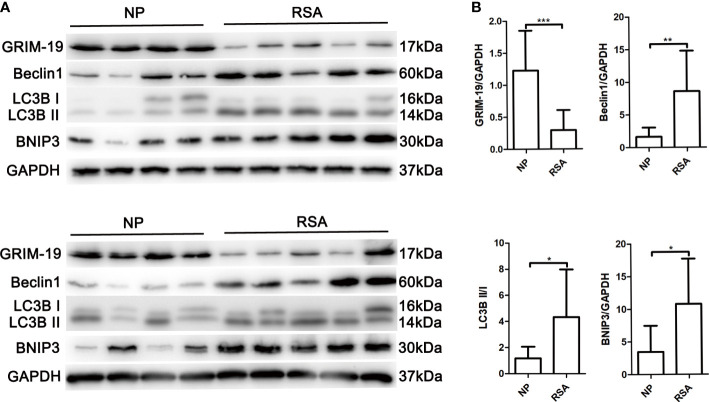
Determination of Grim-19 expression in decidual macrophages of RSA patients and the NP group, and the expression of autophagy related proteins. **(A)** The expression of Grim-19, Beclin1, LC3B II/I and BNIP3 in decidual macrophages of RSA patients (n = 10) and the NP group (n = 8) was examined through western blot analysis. GAPDH served as a loading control. **(B)** Quantification of Grim-19, Beclin1, LC3B II/I and BNIP3 expression through relative densities. Data are shown as the mean ± SD. ***p < 0.001, **p < 0.01, *p < 0.05.

To explore the potential role of Grim-19 in decidual macrophages of RSA patients, the expression of Grim-19 in decidual macrophages of both RSA patients and the NP group was detected. The data showed that compared with the NP group, the expression of Grim-19 was decreased in decidual macrophages of RSA patients (P=0.0008, **Figures 1A, B**). Meanwhile, the protein levels of Beclin1 (P=0.007), LC3B II/I (P=0.0307) and BNIP3 (P=0.0173) were markedly upregulated in the decidual macrophages of RSA patients compared with NP group (**Figures 1A, B**). These results suggested that the decreased expression of Grim-19 in decidual macrophages may be related to the occurrence of RSA.

### *Grim-19* deficiency enhances autophagy in uterine macrophages of *GRIM-19^+/-^* mice

*Grim-19* deficient mice were generated by CRISPR/Cas9-mediated genome engineering. Although the heterozygous mice (*GRIM-19^+/-^*) developed normally and were undistinguished from WT mice by appearance, *Grim-19* null embryos (*GRIM-19* ^-/-^) failed to undergo gastrulation at E7.5 and early organogenesis at E8.5, and died in the early stage of embryonic development (day 9.5) (13). For this reason, the embryo resorption rate of *GRIM-19^+/-^* mice cannot be accurately observed.

**Figure 2:**
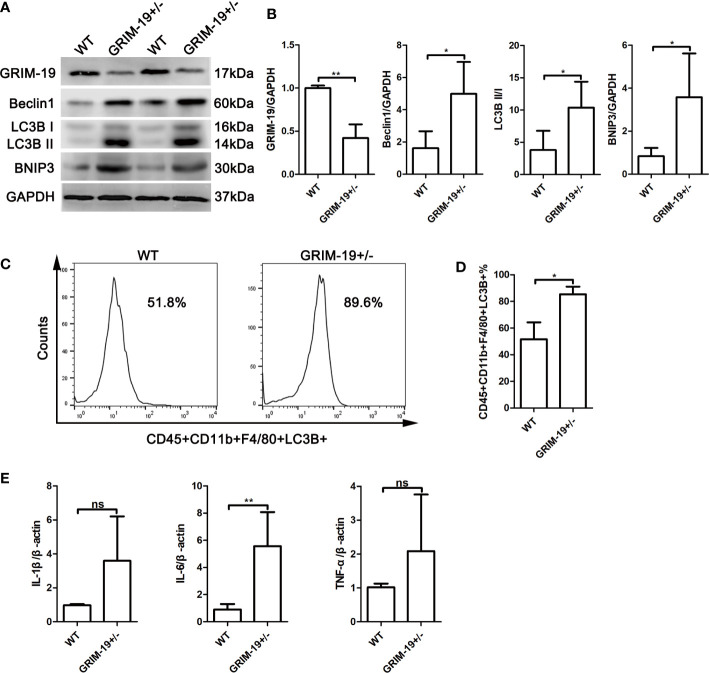
Increase in autophagy and inflammatory factors in uterine mononuclear cells of *GRIM-19^+/-^* mice. **(A)** The expression of GRIM-19, Beclin1, LC3B II/I and BNIP3 in uterine mononuclear cells of *GRIM-19^+/-^* mice and WT mice was examined through western blot analysis (4 mice per group). GAPDH served as a loading control. Three independent experiments were carried out. **(B)** Quantification of GRIM-19, Beclin1, LC3B II/I and BNIP3 expression through relative densities. **(C)** Representative images of flow cytometry to determine LC3B expression in uterine macrophages (CD45+CD11b+F4/80+LC3B+) of *GRIM-19^+/-^* mice and WT mice (4 mice per group). Three independent experiments were carried out. **(D)** Percentages of CD45+CD11b+F4/80+LC3B+ cells were identified. **(E)** The mRNA levels of the proinflammatory cytokines IL1B, IL6 and TNFa in uterine mononuclear cells of *GRIM-19^+/-^* mice and WT mice were detected by quantitative real-time PCR (4 mice per group). Control (WT) mice used in the study were littermates of *GRIM-19^+/-^* mice. Three independent experiments were carried out. Data are shown as the mean ± SD. **p < 0.01, *p < 0.05, ns, not significant.

To determine whether *Grim-19* suppression affects autophagy in immune cells, we isolated uterine mononuclear cells from *GRIM-19^+/-^* mice and evaluated the expression of autophagy associated proteins using western blotting. Results depicted in **Figures 2A, B** showed that downregulation of GRIM-19 (P=0.0071) increased the expression of Beclin1 (P=0.0351), LC3B II/I (P=0.0391), and BNIP3 (P=0.0379) in uterine mononuclear cells of *GRIM-19^+/-^* mice. Then, flow cytometric analysis was used to detect the expression level of LC3B in uterine macrophages of *GRIM-19^+/-^* mice, and a similar result was observed. The proportion of CD45+CD11b+F4/80+LC3B+ cells in *GRIM-19^+/-^* mouse uteri was significantly higher than that in WT mouse uteri (P=0.0135, **Figures 2C, D**). These data collectively demonstrated that GRIM-19 deficiency enhances macrophage autophagy.

### *Grim-19* deficiency promotes the expression of inflammatory factors in uterine mononuclear cells of *GRIM-19^+/-^* mice

Although the etiology of RSA is unknown, it is recognized that an immune disorder is involved. To determine whether GRIM-19 induces inflammatory cytokine expression in uterine immune cells, the expression of the proinflammatory cytokines IL1B, IL6 and TNFa in uterine mononuclear cells of *GRIM-19^+/-^* mice was detected. As shown by the results of quantitative real-time PCR, downregulation of GRIM-19 increased the expression of IL1B (P=0.0552), IL6 (P=0.0034) and TNFa (P=0.1896) in uterine mononuclear cells of *GRIM-19^+/-^* mice, and the difference in IL6 expression was statistically significant (**Figure 2E**).

### Downregulation of GRIM-19 stimulates autophagy in murine macrophage RAW264.7 cells and THP-1-derived macrophages

**Figure 3:**
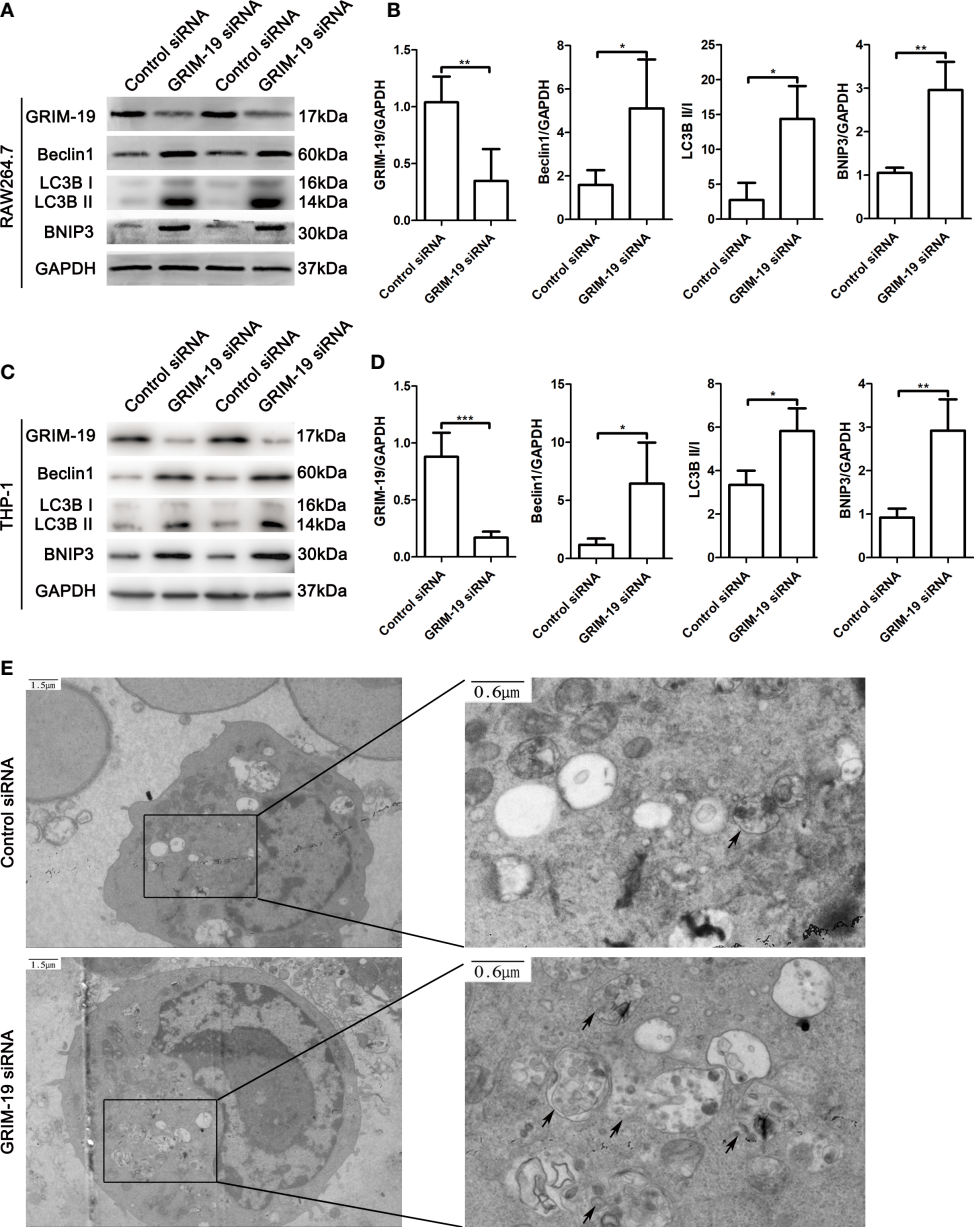
Downregulation of GRIM-19 stimulates autophagy in murine macrophage RAW264.7 cells and THP-1-derived macrophages. **(A)** Representative western blotting images of GRIM-19, Beclin1, LC3B II/I and BNIP3 in RAW264.7 cells after downregulation of GRIM-19. GAPDH served as a loading control. **(B)** Quantification of GRIM-19, Beclin1, LC3B II/I and BNIP3 expression through relative densities. Data are shown as the mean ± SD. Three independent experiments were carried out. **(C)** Representative western blotting images of Grim-19, Beclin1, LC3B II/I and BNIP3 in THP-1 cells after downregulation of Grim-19. GAPDH served as a loading control. **(D)** Quantification of Grim-19, Beclin1, LC3B II/I and BNIP3 expression through relative densities. Data are shown as the mean ± SD. Three independent experiments were carried out. ***p < 0.001, **p < 0.01, *p < 0.05. **(E)** Autophagosomes were observed by TEM.

To investigate the potential biological effect of GRIM-19 on macrophages *in vitro*, we altered GRIM-19 expression by siRNA and determined the level of autophagy in murine macrophage RAW264.7 cells. The transfection efficacy was confirmed using western blot analysis. As depicted in **Figures 3A, B**, downregulation of GRIM-19 (P=0.0086) created a remarkable increase in the expression of Beclin1 (P=0.0244), LC3B II/I (P=0.0191) and BNIP3 (P=0.0012) in RAW264.7 cells. To validate the results in both mouse and human cell lines, we downregulated Grim-19 expression by siRNA in a human macrophage cell line (THP-1-derived macrophages). As shown in the **Figures 3C, D**, downregulation of Grim-19 (P=0.0006) significantly increased the expression of Beclin1 (P=0.0258), LC3B II/I (P=0.0258) and BNIP3 (P=0.0017) in THP-1-derived macrophages. We further evaluated the autophagy flux in GRIM-19 siRNA treated RAW264.7 cells through TEM analysis. As expected, inhibition of GRIM-19 enhanced the formation of autophagosomes (**Figure 3E**). These data collectively demonstrated that downregulation of GRIM-19 stimulated autophagy.

### Downregulation of GRIM-19 in RAW264.7 cells promotes the release of proinflammatory cytokines and phagocytic activity, which can be reversed by autophagy blockade

**Figure 4:**
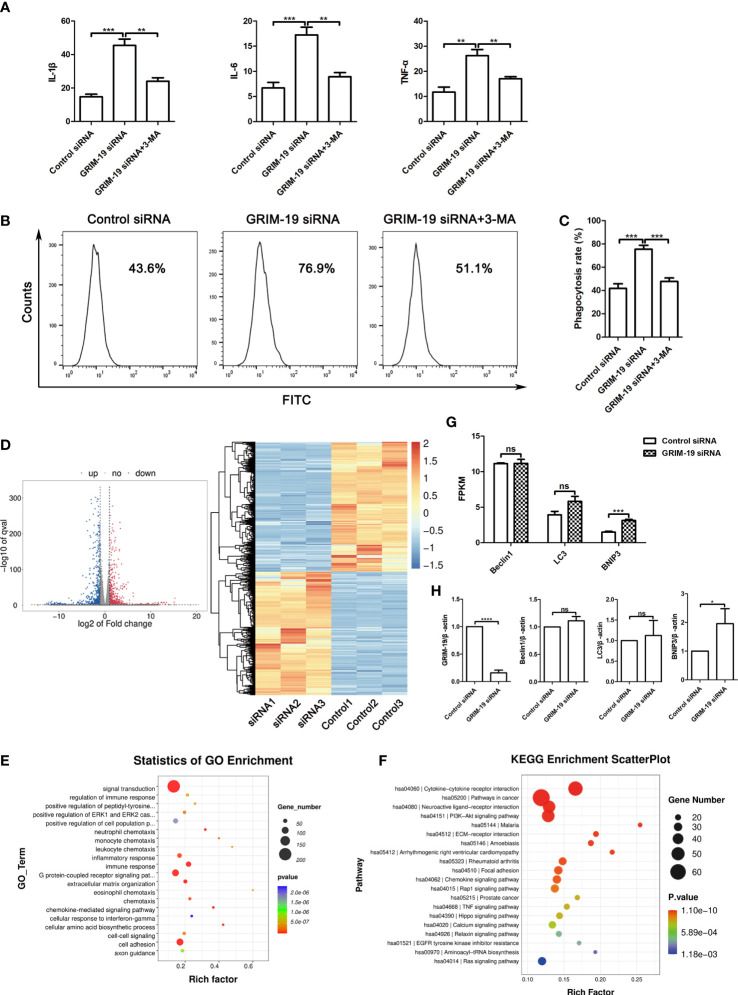
Downregulation of GRIM-19 promotes the release of proinflammatory cytokines and phagocytic activity, which can be reversed by autophagy blockade. **(A)** ELISA analysis of the proinflammatory cytokines IL1B, IL6 and TNFa in the supernatant of RAW264.7 cells in the control siRNA, GRIM-19 siRNA, and GRIM-19 siRNA + 3MA groups. Data are shown as the mean ± SD. Three independent experiments were carried out. **(B)** The intracellular FITC-dextran was measured by flow cytometric analysis in the control siRNA, GRIM-19 siRNA, and GRIM-19 siRNA + 3MA groups. The representative examples are shown. **(C)** The phagocytosis rate in the control siRNA, GRIM-19 siRNA, and GRIM-19 siRNA + 3MA groups was identified. Three independent experiments were carried out. Data are shown as the mean ± SD. **(D)** Volcano plot and cluster analysis of differentially expressed genes from RNA-seq data in Grim-19 and control siRNA treated THP-1-derived macrophages. **(E)** The enriched Gene Ontology terms of biological process categories of differentially expressed genes from RNA-seq data in Grim-19 and control siRNA treated THP-1-derived macrophages. **(F)** KEGG analysis of differentially expressed genes from RNA-seq data in Grim-19 and control siRNA treated THP-1-derived macrophages. **(G)** The expression levels of Beclin1, LC3, and BNIP3 at the transcriptome level were quantified as FPKMs in RNA-seq. The differential gene expression was identified using DESeq2 software. The genes with the parameter of false discovery rate (FDR) below 0.05 and absolute fold change ≥ 2 were considered differentially expressed genes (|log_2_FC|≥1 & q<0.05). **(H)** The mRNA levels of the Beclin1, LC3, and BNIP3 were detected by quantitative real-time PCR. ****p < 0.0001, ***p < 0.001, **p < 0.01, *p < 0.05, ns, not significant.

Dysfunctional autophagy is thought to be a contributing factor in many chronic inflammatory diseases (14). To elucidate the mechanism of GRIM-19 deficiency in macrophages, we explored whether autophagy participates in the influence of GRIM-19 on the release of proinflammatory cytokines in RAW264.7 cells. ELISA analysis of the proinflammatory cytokines in the supernatant revealed that GRIM-19 knockdown led to an increase in the release of IL1B (P=0.0002), IL6 (P=0.0007) and TNFa (P=0.0014) in RAW264.7 cells (**Figure 4A**). However, these effects were effectively reversed by the autophagy inhibitor 3MA. The results depicted in **Figure 4A** show that the introduction of 3MA obviously diminished the effect of GRIM-19 downregulation on IL1B (P=0.001), IL6 (P=0.0012) and TNFa (P=0.0037) expression in RAW264.7 cells.

It has also been reported that decidual macrophages play a role in the phagocytosis of apoptotic trophoblasts (15). To verify the involvement of GRIM-19 in the phagocytic ability of macrophages, phagocytic activity was determined after GRIM-19 knockdown. As shown by the results of flow cytometric analysis, knockdown of GRIM-19 enhanced the ability of macrophages to phagocytose FITC-dextran (P=0.0003, **Figure 4B**). In addition, the introduction of the autophagy inhibitor 3MA effectively decreased the elevated phagocytic activity that was originally induced by GRIM-19 knockdown (P=0.0004, **Figures 4B, C**). These results suggested that GRIM-19 deficiency influenced macrophage function, characterized by enhanced proinflammatory cytokines and phagocytic activity, and this might be regulated by autophagy.

### GRIM-19 is involved in the inflammatory response in THP-1-derived macrophages

To study the effect of downregulation of GRIM-19 on the gene expression of human macrophages, we treated THP-1-derived macrophages with GRIM-19 siRNA and then prepared an RNA sequencing (RNA-seq) library. Transcriptome analysis results showed that 980 genes were downregulated and 914 genes were upregulated (**Figure 4D**). The enrichment and Gene Ontology (GO) analyses showed that the changed genes were enriched in many immune response terms, including regulation of immune response, inflammatory response, immune response, neutrophil, monocyte, leukocyte, and eosinophil chemotaxis (**Figure 4E**). The KEGG analysis of these hub genes was also enriched in many immune and inflammatory pathways, including cytokine-cytokine receptor interaction, rheumatoid arthritis, chemokine signaling pathway, TNF signaling pathway (**Figure 4F**). These results indicate that GRIM-19 mainly modulates immune and inflammatory related pathways, leading to cytokine production, and thus contributing to inflammation.

To verify the role of GRIM-19 in autophagy, we analyzed the changes of LC3, Beclin1, and BNIP3 at the transcriptome level in RNA-seq. The expression level of a certain gene was quantified as FPKMs. Our results showed that BNIP3 expression was increased in GRIM-19 siRNA treated THP-1-derived macrophages, whereas the expression of LC3 and Beclin1 did not change (**Figure 4G**). Next, quantitative real-time PCR was performed to further confirm the RNA-seq results. Our results showed that BNIP3 mRNA expression was increased significantly with downregulation of GRIM-19 in THP-1-derived macrophages, whereas expression of Beclin1 and LC3 were increased but not statistically different (**Figure 4H**).

## Discussion

Macrophages are closely related to homeostatic and tolerogenic properties and have been shown to play important roles in the maintenance of pregnancy (1, 16). However, our understanding of the underlying mechanism of macrophages in pregnancy-associated diseases remains limited. In the present study, we confirmed that Grim-19 downregulation is accompanied by enhanced macrophage autophagy in the decidua of RSA patients.

Previous studies have reported the function of Grim-19 in abortion. Low expression of Grim-19 in the villus was associated with the occurrence of missed abortion by altering angiogenesis and increasing apoptosis (17). Our previous research confirmed that Grim-19 deficiency regulated the Treg/Th17 balance partly through the reactive oxygen species (ROS)-mammalian target of rapamycin (mTOR) signaling axis in unexplained RSA (18). In addition, GRIM-19 appears to regulate innate and acquired immune responses (19). GRIM-19 ameliorates multiple sclerosis through reciprocal regulation of IFNγ/Th1 and IL-17A/Th17 cells in a mouse model of experimental autoimmune encephalomyelitis (20). Studies have also reported that GRIM19 inhibits obesity by regulating Th17/Treg balance and inflammatory white fat browning (21). GRIM19 has been shown to attenuate acute graft-versus-host disease by inhibiting excessive inflammatory response mediated by T cell activation (22). Our study reported that Grim-19 played a role in RSA by regulating macrophage function.

Cumulative evidence suggests that Grim-19 is correlated with autophagy, although the underlying molecular mechanism remains unclear. In Hela cells, Grim-19 inhibition was shown to induce autophagy through activation of HIF-1α and ERK (23). More recently, a study also reported that Grim-19 repressed hypoxia-induced invasion and epithelial-mesenchymal transition by repressing autophagy through inactivation of the STAT3/HIF-1α signaling axis in colorectal cancer (12). In line with previous studies, we confirmed that GRIM-19 deficiency induces macrophage autophagy in *GRIM-19^+/-^* mouse uteri. However, because both embryonic and maternal factors are involved, we cannot accurately observe the embryonic resorption rate of *GRIM-19^+/-^* mice, which is a shortcoming of this study.

The dysfunction of macrophages is involved in pregnancy disorders, such as RSA, infertility, preeclampsia, intrauterine growth restriction, and preterm labor (24). Mammalian decidual macrophages have attracted widespread attention due to their wide variety of functions, such as (a) production of cytokines, (b) phagocytosis and tissue remodeling, (c) defense against infections, and (d) affect the function of surrounding cells like endothelial cells of arteries, glandular epithelium, and invasive cytotrophoblasts (24–27). Although accumulating evidence has confirmed the important roles of macrophages in pregnancy related diseases, the molecular mechanisms underlying macrophage dysfunction remain poorly understood.

During the complex process of pregnancy, macrophages play crucial roles in the maintenance of maternal-fetal tolerance by secreting many cytokines (24). Inappropriate immune and inflammatory responses have been confirmed to be associated with unexplained RSA (4, 28, 29). Studies have shown that increased expression of IL1B in the villi and decidua easily induces adverse pregnancy outcomes (30). Studies have also reported that inflammatory cytokines cervical IL6 and IL-8 produced by macrophages might have predictive value in cases of recurrent miscarriage (31). Our data in this study showed that GRIM-19 deficiency promotes the expression of inflammatory factors in uterine macrophages of *GRIM-19^+/-^* mice, and downregulation of GRIM-19 in RAW264.7 cells also promotes the release of proinflammatory cytokines. Therefore, it is not difficult to speculate that when GRIM-19 is downregulated in macrophages, the secretion of inflammatory cytokines can be enhanced, resulting in abnormal inflammatory reactions at the maternal-fetal interface.

Autophagy plays important roles in multiple biological functions, including inflammatory responses. Administration of 3MA (an autophagy inhibitor) attenuated IL6 and TNFa production as well as sepsis symptoms in a lethal model of murine sepsis (5, 32). The results of our study illustrated that the introduction of 3MA effectively decreased the elevated IL1B, IL6 and TNFa expression that was originally induced by GRIM-19 inhibition. However, the role of autophagy in cytokine production involves more specific mechanisms.

Macrophages are large vacuolar cells with high phagocytic capacity. During the process of pregnancy, macrophages phagocytose apoptotic cells, promote spiral artery remodeling and trophoblast invasion, and provide a balanced microenvironment at the maternal-fetal interface (15, 33). Macrophages produce type-I cytokines, such as TNFa, IFN-γ, and IL-12, after phagocytosis of aponecrotic and necrotic trophoblasts and aggravate inflammation (34). Phagocytosis and autophagy in macrophage shared lots of genes, such as Atg5, Vps34, and Beclin1 (5, 35). IFN-α and IL1B production has also been verified to rely on LC3-associated phagocytosis in TLR9-related tracking (36). Our data suggested that knockdown of GRIM-19 enhanced the phagocytic activity of macrophages, and autophagy suppression by the autophagy inhibitor 3MA overcame, at least partially, that enhanced phagocytic activity.

In conclusion, our findings provide evidence supporting the notion that Grim-19 deficiency influences macrophage function, characterized by enhanced proinflammatory cytokines and phagocytic activity, and the process is mediated through autophagy. These factors together trigger excessive inflammatory response at the maternal-fetal interface and play an important role in the occurrence of RSA.

## Data availability statement

The data presented in the study are deposited in the NCBI repository, accession number PRJNA891625.

## Ethics statement

The studies involving human participants were reviewed and approved by Medical Ethics Committee of Qilu Hospital of Shandong University. The patients/participants provided their written informed consent to participate in this study. The animal study was reviewed and approved by Medical Ethics Committee of Qilu Hospital of Shandong University.

## Author contributions

AH and YY conceived the study. YY, HL, YZ and CG performed experiments and data analysis. AH, YY and LC wrote, reviewed, and edited the manuscript. All authors edited and approved the final draft of the manuscript.

## Funding

This work was supported by the National Natural Science Foundation of China (grant numbers 81701528, 82071620 and 82001638).

## Conflict of interest

The authors declare that the research was conducted in the absence of any commercial or financial relationships that could be construed as a potential conflict of interest.

## Publisher’s note

All claims expressed in this article are solely those of the authors and do not necessarily represent those of their affiliated organizations, or those of the publisher, the editors and the reviewers. Any product that may be evaluated in this article, or claim that may be made by its manufacturer, is not guaranteed or endorsed by the publisher.

## References

[1] Zhu X Liu H Zhang Z Wei R Zhou X Wang Z. Mir-103 protects from recurrent spontaneous abortion *Via* inhibiting Stat1 mediated M1 macrophage polarization. Int J Biol Sci (2020) 16(12):2248–64. doi: 10.7150/ijbs.46144 PMC7294935 32549769

[2] Ticconi C Pietropolli A Di Simone N Piccione E Fazleabas A. Endometrial immune dysfunction in recurrent pregnancy loss. Int J Mol Sci (2019) 20(21):5332. doi: 10.3390/ijms20215332 PMC6862690 31717776

[3] Ying X Jin X Zhu Y Liang M Chang X Zheng L. Exosomes released from decidual macrophages deliver mir-153-3p, which inhibits trophoblastic biological behavior in unexplained recurrent spontaneous abortion. Int Immunopharmacol (2020) 88:106981. doi: 10.1016/j.intimp.2020.106981 33182030

[4] Gao P Zha Y Gong X Qiao F Liu H. The role of maternal-foetal interface inflammation mediated by Nlrp3 inflammasome in the pathogenesis of recurrent spontaneous abortion. Placenta (2020) 101:221–9. doi: 10.1016/j.placenta.2020.09.067 33022545

[5] Wu MY Lu JH. Autophagy and macrophage functions: Inflammatory response and phagocytosis. Cells (2019) 9(1):70. doi: 10.3390/cells9010070 PMC7016593 31892110

[6] Wang WJ Hao CF Lin QD. Dysregulation of macrophage activation by decidual regulatory T cells in unexplained recurrent miscarriage patients. J Reprod Immunol (2011) 92(1-2):97–102. doi: 10.1016/j.jri.2011.08.004 22015003

[7] Liu K Zhao E Ilyas G Lalazar G Lin Y Haseeb M. Impaired macrophage autophagy increases the immune response in obese mice by promoting proinflammatory macrophage polarization. Autophagy (2015) 11(2):271–84. doi: 10.1080/15548627.2015.1009787 PMC4502775 25650776

[8] Qiu P Liu Y Zhang J. Review: The role and mechanisms of macrophage autophagy in sepsis. Inflammation (2019) 42(1):6–19. doi: 10.1007/s10753-018-0890-8 30194660

[9] Lu H Cao X. Grim-19 is essential for maintenance of mitochondrial membrane potential. Mol Biol Cell (2008) 19(5):1893–902. doi: 10.1091/mbc.e07-07-0683 PMC2366854 18287540

[10] Moreira S Correia M Soares P Máximo V. Grim-19 function in cancer development. Mitochondrion (2011) 11(5):693–9. doi: 10.1016/j.mito.2011.05.011 21664299

[11] Moon YM Lee J Lee SY Her YM Ryu JG Kim EK. Gene associated with retinoid-Interferon-Induced mortality 19 attenuates murine autoimmune arthritis by regulation of Th17 and treg cells. Arthritis Rheumatol (2014) 66(3):569–78. doi: 10.1002/art.38267 24574216

[12] Zhang J Chu D Kawamura T Tanaka K He S. Grim-19 repressed hypoxia-induced invasion and emt of colorectal cancer by repressing autophagy through inactivation of Stat3/Hif-1α signaling axis. J Cell Physiol (2019) 234(8):12800–8. doi: 10.1002/jcp.27914 PMC13483207 30537081

[13] Huang G Lu H Hao A Ng DC Ponniah S Guo K. Grim-19, a cell death regulatory protein, is essential for assembly and function of mitochondrial complex I. Mol Cell Biol (2004) 24(19):8447–56. doi: 10.1128/mcb.24.19.8447-8456.2004 PMC516758 15367666

[14] Iida T Onodera K Nakase H. Role of autophagy in the pathogenesis of inflammatory bowel disease. World J Gastroenterol (2017) 23(11):1944–53. doi: 10.3748/wjg.v23.i11.1944 PMC5360635 28373760

[15] Straszewski-Chavez SL Abrahams VM Mor G. The role of apoptosis in the regulation of trophoblast survival and differentiation during pregnancy. Endocrine Rev (2005) 26(7):877–97. doi: 10.1210/er.2005-0003 15901666

[16] Mor G Aldo P Alvero AB. The unique immunological and microbial aspects of pregnancy. Nat Rev Immunol (2017) 17(8):469–82. doi: 10.1038/nri.2017.64 28627518

[17] Chen H Deng X Yang Y Shen Y Chao L Wen Y. Expression of grim-19 in missed abortion and possible pathogenesis. Fertility sterility (2015) 103(1):138–46.e3. doi: 10.1016/j.fertnstert.2014.10.012 25455534

[18] Yang Y Cheng L Deng X Yu H Chao L. Expression of grim-19 in unexplained recurrent spontaneous abortion and possible pathogenesis. Mol Hum Reprod (2018) 24(7):366–74. doi: 10.1093/molehr/gay020 29741731

[19] Nallar SC Kalvakolanu DV. Grim-19: A master regulator of cytokine induced tumor suppression, metastasis and energy metabolism. Cytokine Growth factor Rev (2017) 33:1–18. doi: 10.1016/j.cytogfr.2016.09.001 27659873 PMC5337140

[20] Moon J Lee SH Lee SY Ryu J Jhun J Choi J. Grim-19 ameliorates multiple sclerosis in a mouse model of experimental autoimmune encephalomyelitis with reciprocal regulation of Ifnγ/Th1 and il-17a/Th17 cells. Immune network (2020) 20(5):e40. doi: 10.4110/in.2020.20.e40 33163248 PMC7609166

[21] Jhun J Woo JS Lee SH Jeong JH Jung K Hur W. Grim19 impedes obesity by regulating inflammatory white fat browning and promoting Th17/Treg balance. Cells (2021) 10(1):162. doi: 10.3390/cells10010162 33467683 PMC7829987

[22] Park MJ Lee SH Lee SH Kim EK Lee EJ Moon YM. Grim19 ameliorates acute graft-Versus-Host disease (Gvhd) by modulating Th17 and treg cell balance through down-regulation of Stat3 and nf-at activation. J Trans Med (2016) 14(1):206. doi: 10.1186/s12967-016-0963-0 PMC4938933 27391226

[23] Yue X Zhao P Wu K Huang J Zhang W Wu Y. Grim-19 inhibition induced autophagy through activation of erk and hif-1α not Stat3 in hela cells. Tumour Biol (2016) 37(7):9789–96. doi: 10.1007/s13277-016-4877-5 26810068

[24] Jena MK Nayak N Chen K Nayak NR. Role of macrophages in pregnancy and related complications. Archivum immunol therapiae experimentalis (2019) 67(5):295–309. doi: 10.1007/s00005-019-00552-7 PMC7140981 31286151

[25] Laskarin G Cupurdija K Tokmadzic VS Dorcic D Dupor J Juretic K. The presence of functional mannose receptor on macrophages at the maternal-fetal interface. Hum Reprod (Oxford England) (2005) 20(4):1057–66. doi: 10.1093/humrep/deh740 15746201

[26] Heikkinen J Möttönen M Komi J Alanen A Lassila O. Phenotypic characterization of human decidual macrophages. Clin Exp Immunol (2003) 131(3):498–505. doi: 10.1046/j.1365-2249.2003.02092.x 12605704 PMC1808648

[27] Mor G Abrahams VM. Potential role of macrophages as immunoregulators of pregnancy. Reprod Biol Endocrinol (2003) 1:119. doi: 10.1186/1477-7827-1-119 14651752 PMC305335

[28] Bulla R Bossi F Tedesco F. The complement system at the embryo implantation site: Friend or foe? Front Immunol (2012) 3:55. doi: 10.3389/fimmu.2012.00055 22566936 PMC3341982

[29] Chaouat G. The Th1/Th2 paradigm: Still important in pregnancy? Semin immunopathol (2007) 29(2):95–113. doi: 10.1007/s00281-007-0069-0 17626305

[30] Saji F Samejima Y Kamiura S Sawai K Shimoya K Kimura T. Cytokine production in chorioamnionitis. J Reprod Immunol (2000) 47(2):185–96. doi: 10.1016/s0165-0378(00)00064-4 10924750

[31] Hattori Y Nakanishi T Ozaki Y Nozawa K Sato T Sugiura-Ogasawara M. Uterine cervical inflammatory cytokines, interleukin-6 and -8, as predictors of miscarriage in recurrent cases. Am J Reprod Immunol (New York NY: 1989) (2007) 58(4):350–7. doi: 10.1111/j.1600-0897.2007.00516.x 17845205

[32] Li Q Li L Fei X Zhang Y Qi C Hua S. Inhibition of autophagy with 3-methyladenine is protective in a lethal model of murine endotoxemia and polymicrobial sepsis. Innate Immun (2018) 24(4):231–9. doi: 10.1177/1753425918771170 PMC6830927 29673286

[33] Yao Y Xu XH Jin L. Macrophage polarization in physiological and pathological pregnancy. Front Immunol (2019) 10:792. doi: 10.3389/fimmu.2019.00792 31037072 PMC6476302

[34] Huppertz B Kingdom J Caniggia I Desoye G Black S Korr H. Hypoxia favours necrotic versus apoptotic shedding of placental syncytiotrophoblast into the maternal circulation. Placenta (2003) 24(2-3):181–90. doi: 10.1053/plac.2002.0903 12566245

[35] Huang H Li X Zhuang Y Li N Zhu X Hu J. Class a scavenger receptor activation inhibits endoplasmic reticulum stress-induced autophagy in macrophage. J Biomed Res (2014) 28(3):213–21. doi: 10.7555/jbr.28.20130105 PMC4085558 25013404

[36] Henault J Martinez J Riggs JM Tian J Mehta P Clarke L. Noncanonical autophagy is required for type I interferon secretion in response to DNA-immune complexes. Immunity (2012) 37(6):986–97. doi: 10.1016/j.immuni.2012.09.014 PMC3786711 23219390

